# Rethinking the Niche of Upper-Atmosphere Bacteria: Draft Genome Sequences of *Bacillus aryabhattai* C765 and *Bacillus aerophilus* C772, Isolated from Rice Fields

**DOI:** 10.1128/genomeA.00094-15

**Published:** 2015-04-09

**Authors:** Paula Ramos-Silva, Patrícia H. Brito, Mónica Serrano, Adriano O. Henriques, José B. Pereira-Leal

**Affiliations:** a Instituto Gulbenkian de Ciência, Oeiras, Portugal; b Instituto de Tecnologia Química e Biológica, Universidade Nova de Lisboa, Oeiras, Portugal

## Abstract

Here, we report two genome sequences of endospore-forming bacteria isolated from the rice fields of Comporta, Portugal, identified as *Bacillus aryabhattai* C765 and *Bacillus aerophilus* C772. Both species were previously identified in air samples from the upper atmosphere, but our findings suggest their presence in a wider range of environmental niches.

## GENOME ANNOUNCEMENT

The strains *Bacillus aryabhattai* C765 and *Bacillus aerophilus* C772 were identified in samples collected from the rice fields of the Portuguese coastal area of Comporta, which is near the Sado River and the Atlantic Ocean. This finding confirms the presence of these species in a broader set of environments than was previously suggested (1, 2), supporting a freshwater ecological niche.

When grown in Difco sporulation medium (DSM), both organisms form subterminal ellipsoidal endospores that do not swell the mother cell. Prior to DNA extraction, the cells were grown at 37°C in LB medium until reaching an optical density at 600 nm (OD_600_) of 1. The genomes were sequenced with the Illumina MiSeq system (300-bp paired-end library), generating >1.8 million reads, with average coverages of 123× and 146× for *B. aryabhattai* C765 and *B. aerophilus* C772, respectively. More than 94% of the reads were assembled using SPAdes 3.0.0 (3). A total of 49 contigs (5.5 Mbp, 38% G+C content) were obtained for *B. aryabhattai* C765, and 17 contigs were obtained for *B. aerophilus* (3.7 Mbp, 41% G+C content). Only the contigs of >1,000 bp and with a coverage of >10× were used. In the assembly of *B. aryabhattai*, one contig corresponds to a complete sequence of a plasmid of 10,154 bp, with a coverage of 1,339×. Another five contigs showed high identity (≥88%) and significant overlap (≥18%) with the plasmid sequences of *Bacillus megaterium* QMB1551; still, it was not possible to conclude whether these contigs belong to plasmid or chromosomal DNA.

A total of 5,683 gene predictions in *B. aryabhattai* C765 were generated with Prodigal 2.60 (4) and annotated using Prokka 1.9 (5). The same software predicted 3,809 genes in *B. aerophilus* C772.

Searches in the 16S rRNA database EzTaxon (6) indicated *B. aryabhattai* B8W22^T^ and *B. aerophilus* 28K^T^ as the closest species (>98.6% sequence identity).

Reciprocal Best-Hits BLAST analyses revealed that strain C765 has a surprising 896 strain-specific genes in relation to the closest genomes of *B. aryabhattai* GZ203 and *B. megaterium* WSH-002 (5), and these organisms share a core of 4,361 orthologues. Both strains of *B. aryabhattai* share about 4% more orthologous genes than they do with *B. megaterium*. Strain C772 has 226 strain-specific genes and shares 3,391 orthologous genes with *Bacillus* sp. M 2-6 strain KACC 16563, a possible representative of *B. aerophilus* (6), and *Bacillus pumilus* MTCC B6033 (7). Strain C772 shares 6% more orthologous genes with strain KACC 16563 than it does with *B. pumilus*. In both genomes, the top GO terms (7) of specific genes corresponded to DNA binding (GO:0003677) and ATP binding functions (GO:0005524).

The plasmid identified in *B. aryabhattai* C765, termed pBa1, contains 10 coding sequences (CDSs), including two putative relaxase/mobilization proteins, one 3-oxo-5-alpha-steroid 4-dehydrogenase, one plasmid replication protein (RepL), and one antibiotic resistance regulatory protein of the MarR family.

### Nucleotide sequence accession numbers.

The whole-genome shotgun projects of *B. aryabhattai* C765 and *B. aerophilus* C772 were deposited at DDBJ/EMBL/GenBank under the accession numbers JXRC00000000 and JXRO00000000.
